# Segmental Colitis Associated Diverticulosis—A Possible Diagnosis in Teenagers

**DOI:** 10.3389/fped.2018.00168

**Published:** 2018-06-05

**Authors:** Cristina O. Mǎrginean, Lorena E. Meliţ, Maria O. Mǎrginean

**Affiliations:** Department of Pediatrics I, University of Medicine and Pharmacy of Tîrgu Mureş, Târgu Mureş, Romania

**Keywords:** colonic diverticulosis, ulcerative colitis, segmental colitis, pediatrics, teenager

## Abstract

Segmental colitis associated with diverticulosis (SCAD) is manifested by active chronic inflammation of the colonic segments affected by diverticulosis, luminal-mucosal inflammation, independent of the presence of inflammation within and/or around the diverticula, and it usually spares the rectum. We present the case of a 15-year-old female admitted to our clinic due to lower digestive hemorrhage and abdominal pain in the previous week, associated with fever 1 day prior to admission. The patient had pallor, painful abdomen upon palpation, accelerated bowel movements, and macroscopic evidence of blood in the stools. Initial laboratory tests showed leukocytosis with neutrophilia, thrombocytosis, anemia, and elevated inflammatory biomarkers. Moreover, colonoscopy revealed multiple ulcerations, hemorrhage, and edema of the sigmoid colon; however, multiple orifices raised the suspicion of a colonic diverticulosis and this was later on confirmed through a barium enema. The histopathological examination pointed out signs of an active chronic inflammation and mucosal architectural changes associated with crypt abscesses, therefore suggesting the diagnosis of SCAD. The patient's prognosis was favorable; her condition improved following steroid and 5-aminosalicylate therapy, without any symptoms or recurrences at the 4 months follow-up. In conclusion, SCAD is a very rare disease entity that is usually confused with other inflammatory bowel conditions. Moreover, it has not been reported in the pediatric age group until now.

## Introduction

Diverticula is a condition characterized by herniation of the mucosa and submucosa through defects in the colonic muscular layer at the place of penetration by vasa recta, which presents one of the most frequent anatomic alterations of the colon (1, 2). The incidence of colonic diverticulosis (CD) increases with age, with a 45% prevalence in the population > 70 years of age, but it can also be encountered in patients < 40 years old (3). Inflammatory bowel disease (IBD), which mainly includes ulcerative colitis (UC) and Crohn's disease, is a chronic condition of the gastrointestinal tract and is probably one of the most common diseases affecting children in developed countries (4). Pseudodiverticula or pseudosacculations are defined as mucosal projections through acquired defects of the bowel walls, resulting from inflammatory fibrous tissue or contraction and ulceration of one side of the bowel, which leads to the formation of a redundant sac on the opposite side (5). These disease entities can be encountered in both Crohn's disease and ischemic colitis.

Segmental colitis associated with diverticulosis (SCAD) is represented by an active chronic inflammation at the level of colonic segments affected by diverticulosis. It is manifested by luminal mucosal inflammation, regardless of the presence of inflammation within and/or around the diverticula, and it usually spares the recum (6). Its prevalence is difficult to establish, as people usually lack the awareness of this condition, and its clinical signs overlap with UC or Crohn's disease. Nevertheless, studies reported its prevalence to be 0.25–1.4% in the general population; however, in patients with CD, it varies between 1.15 and 11.4% (6). SCAD can closely mimic UC due to pathological changes that can range from mild nonspecific inflammation to intense active inflammation with crypt abscesses, crypt distortion, and/or even severe chronic inflammation (6). The hypothesized mechanism leading to this condition includes relative ischemia of the sigmoid colon and impairment of the bacterial flora due to stasis (7). The distinction between UC and SCAD is also hindered by other facts. Therefore, SCAD can precede the onset of UC or the possibility of UC with relative rectal sparing, although the latter is uncommon (8–10). In addition, UC can be associated with CD in some cases, although the prevalence of CD has been reported to be lower in patients diagnosed with UC as compared to the general population (11, 12). Therefore, to ease the clinician's task in establishing a proper diagnosis, Tursi et al. tried to define the diagnostic criteria for UC and SCAD (13). Thus, UC with diverticulosis is defined by macroscopic and microscopic evidence of inflammation of the rectal and colonic mucosa associated with diverticulosis in the sigmoid and descending colon and by the evidence of macroscopic and microscopic inflammation of the diverticular ostia. On the other hand, SCAD is represented by macroscopic and microscopic inflammation of the sigmoid and descending colonic mucosa, without diverticular ostia involvement, and it is associated with a normal rectum and proximal colon. Based on endoscopic findings and histopathological changes, SCAD has been divided into four subtypes: type A or crescentic fold; type B/ mild-to-moderate UC-like; type C or Crohn's disease-like; and type D or severe UC-like (14). All these subtypes commonly manifest themselves by diverticular sparing, which is the main distinction between SCAD and IBD. Hence, the distinction between SCAD and UC can be a real challenge for clinicians, regardless of the endoscopic and histopathological findings.

We present this case with the aim of highlighting the fact that SCAD can also be encountered in teenagers and to increase the pediatrician's awareness of this condition.

Informed consent was obtained from the patient's father (legal guardian) for the publication of this case report.

## Case report

### Presenting concerns

We present the case of a 15-year-old female admitted to our clinic due to lower digestive tract hemorrhage and abdominal pain within the previous week, associated with fever 1 day prior to admission. She was initially referred to the Infectious Diseases Clinic, where infectious enterocolitis was excluded based on the negative result of the stool culture, which ruled out infections with *Escherichia coli* spp., *Salmonella* spp., *Shigella* spp., *Klebsiella* spp., and *Clostridium difficile*. She also benefited from a surgical consult, which ruled out a surgical condition. The anamnesis did not reveal any significant elements.

### Clinical findings

Physical examination revealed pallor, accelerated bowel movements, painful abdomen upon palpation, and macroscopic evidence of blood in the stools. The patient weighed 50 kilograms but lost four kilograms within the previous 3 months.

### Diagnostic focus and assessment

**Figure 1:**
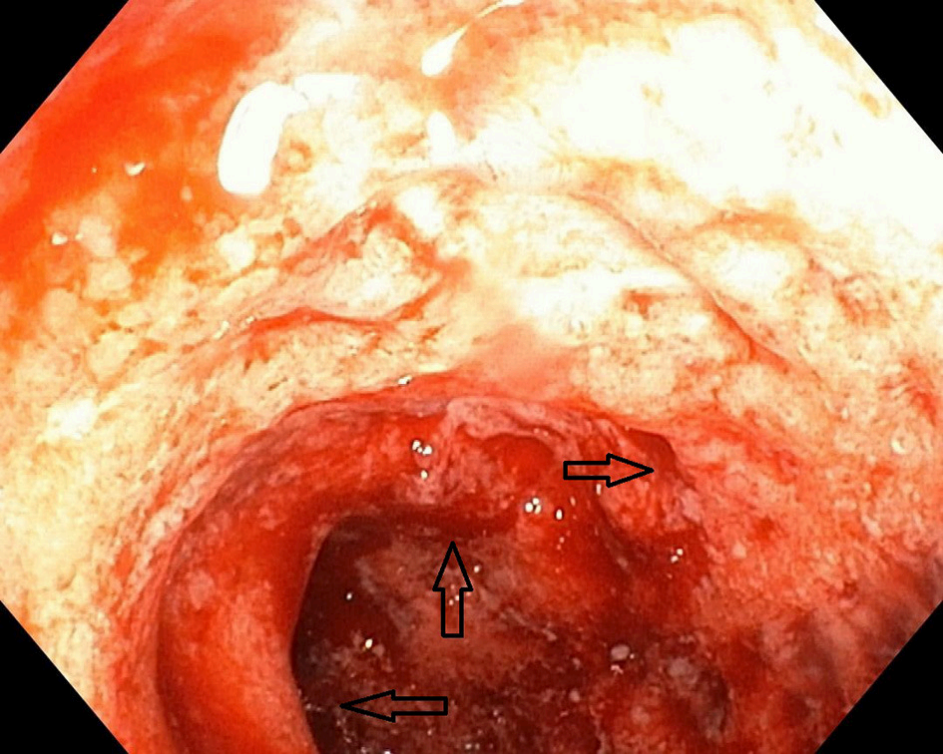
Initial aspect of the colon at colonoscopy.

The laboratory tests performed on the first day of admission revealed leukocytosis with neutrophilia (Leu 15,400/μL; Neu 10,800/μL), thrombocytosis (Plt 527,000/μL), anemia (Hb 9.4 g/dL; Hct 28.3%), elevated inflammatory biomarkers (CRP 26.53 mg/L; ESR 39 mm/h), and low serum urea levels (17.21 mg/dL). The abdominal ultrasound examination was normal. Lower digestive endoscopy pointed out multiple ulcerations, hemorrhage, and edema of the sigmoid colon (Figure 1), thus suggesting a possible IBD; however multiple orifices raised the suspicion of a CD, which was afterwards confirmed through a barium enema.

Histopathological examination of the colonic biopsy specimens showed active inflammation associated with architectural changes of the colonic mucosa and crypt abscesses, which highly suggested a chronic inflammatory process, most likely UC. By taking into account the macroscopic aspect of the colonic mucosa and the histopathological results correlated with the presence of colonic diverticula, we were able to establish the diagnosis of SCAD.

### Therapeutic focus and assessment

**Figure 2:**
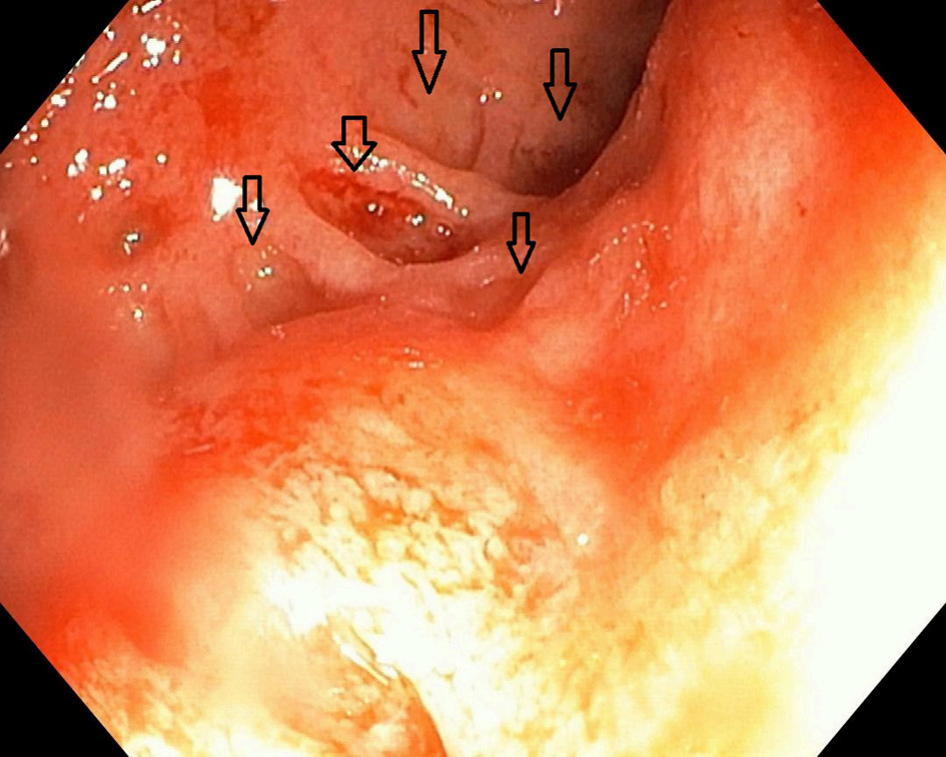
Aspect of the colon after 6 weeks.

We initiated therapy with corticosteroids (Prednisone 1 mg/kg/day, orally) and 5-amino-salicylate derivatives (Mesalazine 50 mg/kg/day, orally), as well supportive treatment (blood transfusions, antibiotics—Ciprofloxacin 20 mg/kg/day orally, glucose, electrolytes, and analgesics). The initial disease progression, within the first 2 weeks, was burdened by the presence of up to 11 bloody stools per day, prompting the patient to undergo multiple blood transfusions (Hb < 7 mg/dL). After ~14 days of corticosteroid therapy, the patient's condition started to progressively improve. We repeated colonoscopy after ~6 weeks (Figure 2), and we observed an improvement of the initial colonic lesions.

### Follow-up and outcome

The patient's prognosis improved within the next 6 months, without any macroscopic signs of lower digestive hemorrhage. Corticosteroid therapy was tapered after 6 weeks, and the patient was given only 5-ASA (mesalazine) instead.

## Discussions

SCAD is a rare clinical entity, defined recently, and it is usually found in the elderly, affecting almost exclusively people > 50 years of age, especially males (15, 16). Contrariwise, our case presented a potential diagnosis of SCAD in a 15-year-old female; hence, the mechanism of SCAD is not yet fully understood. The clinical picture of SCAD is nonspecific, and it is characterized by rectal bleeding (fresh or altered blood), chronic diarrhea, abdominal pain, and less often systemic features such as fever and weight loss (6, 17). Moreover, most patients present more than one symptom (6). Our patient presented rectal bleeding, and associated fever. Laboratory tests are usually useful only for the differential diagnosis, because most often, all parameters are within the reference range, with leukocytosis being rarely reported (17). Nonetheless, laboratory findings pointed out leukocytosis, mild thrombocytosis, anemia, and mildly elevated inflammatory biomarkers (CRP and ESR) in our patient. Unlike the natural undulant course of IBD with multiple relapses and remissions, SCAD has a more benign clinical course. Therefore, a significant proportion of these patients recover completely without any treatment or recurrence (15). Amongst the four subtypes of SCAD described in literature, UC-like changes (types B and D) present a higher risk of relapse, therefore medical treatment should be strongly considered in these cases (18). Similarly, our patient presented SCAD with UC-like changes and her prognosis was slowly favorable with severe rectal bleeding despite therapy with steroids, antibiotics, and salicylates (mesalazine). Fortunately, patients diagnosed with SCAD usually respond very well to an oral 5-aminosalicylate, but in cases with persistent chronically active and symptomatic disease, steroid therapy and/or surgical therapy might be required (16). Although our patient was initially burdened by severe rectal bleeding, which prompted multiple blood transfusions, after ~2 weeks of oral steroids and 5-aminosalicylate, her symptomatology considerably improved, without any signs of relapse after 6 months.

SCAD can also be misdiagnosed as acute uncomplicated diverticulitis. These three clinical entities, namely SCAD, UC, and acute uncomplicated diverticulitis, are represented by inflammatory changes of the colonic mucosa that might impair the same colonic segments. Usually, in acute diverticulitis, the inflammation mainly includes the colonic mucosa surrounding the diverticula, the inflammatory process being largely extended only in severe cases (13). The same study underlined the incidence of UC with diverticulosis and SCAD and acute uncomplicated diverticulitis to be 0.3 and 2%, respectively (13).

Diverticular bleeding is one of the most frequent complications of CD. In up to 90% of cases, colonic diverticular bleeding is resolved spontaneously (19, 20). Nevertheless, ~4% of these patients experience severe symptomatology (21).

Even though it is clear that SCAD and UC are very difficult to be distinguished regardless of the availability of endoscopic and histopathological examimations and as these conditions often present the same symptoms, one must remember that SCAD can precede UC or UC with relative rectal sparing (6, 10).

Pseudodiverticula represent another clinical entity that must be differentiated from SCAD. It is a false diverticula with the formation of a redundant sac from a chronic inflammatory process or contraction and ulceration of the bowel walls. Pseudodiverticula associated with Crohn's disease can appear in any segment of the colon, whereas ischemic pseudodiverticula tends to develop mostly within the splenic flexure (5). In the case presented above, the diverticula were present along the descending colon, and therefore, the diagnosis was more inclined toward SCAD than ischemic pseudodiverticula as a result of severe UC.The severe inflammatory process was confirmed by the macroscopic aspect noticed at colonoscopy, and our patient did not present any previous symptoms. Contrariwise, a long-term severe inflammatory process would have required for pseudodiverticula to develop and to be most likely associated with clinical symptoms. All these aspects combined also suggest that the appropriate diagnosis for our patient should be SCAD. Furthermore, in patients diagnosed with ischemic colitis, hemodynamically significant hemorrhage is uncommon (22); in contrast, our patient needed multiple blood transfusions at the moment of initial diagnosis.

According to a meta-analysis on 486 SCAD cases, the youngest patient reported to be affected was 26 years old (23). Therefore, it is essential to emphasize that our case, to our knowledge, is the first to describe a potential diagnosis of SCAD in teenagers.

## Conclusions

Despite the severe onset of the disease, once we established the diagnosis and administered adequate treatment, the patient's condition improved outstandingly. Therefore, even though SCAD can be a life-threatening condition in cases of diagnosis delay, physician's awareness is essential for a good patient outcome. Increased awareness amongst pediatricians can lead to a proper diagnosis and can lower the risk of misdiagnosing SCAD.

## Table of contents summary

Segmental colitis associated with diverticulosis (SCAD) can also be present in children and to increase the pediatrician's awareness in order to establish a proper diagnosis.

## Author contributions

COM and LM conceptualized and designed the study, drafted the initial manuscript, and reviewed and revised the manuscript. MOM designed the data collection instruments, collected data, carried out the initial analyses, and reviewed and revised the manuscript. All authors approved the final manuscript as submitted and agree to be accountable for all aspects of the work.

### Conflict of interest statement

The authors declare that the research was conducted in the absence of any commercial or financial relationships that could be construed as a potential conflict of interest.

## Abbreviations

Hb: hemoglobin
Htc: hematocrit
CD: colonic diverticulosis
CRP: C-reactive protein
ESR: erythrocyte sedimentation rate
IBD: inflammatory bowel disease
Leu: leukocytes
Neu: neutrophils
Plt: platelets
SCAD: segmental colitis associated with diverticulosis
UC: ulcerative colitis.
